# Evolutionary and Functional Analysis of Coagulase Positivity among the Staphylococci

**DOI:** 10.1128/mSphere.00381-21

**Published:** 2021-08-04

**Authors:** Amy C. Pickering, Gonzalo Yebra, Xiangyu Gong, Mariya I. Goncheva, Bryan A. Wee, Alison C. MacFadyen, Lukas F. Muehlbauer, Joana Alves, Robyn A. Cartwright, Gavin K. Paterson, J. Ross Fitzgerald

**Affiliations:** a The Roslin Institute and Edinburgh Infectious Diseases, University of Edinburgh, Easter Bush, Midlothian, Scotland, United Kingdom; University of Nebraska Medical Center

**Keywords:** *Staphylococcus*, coagulase, coagulase-positive staphylococci, coagulation, diagnostics, evolution, host specificity, phylogeny, von Willebrand binding protein

## Abstract

The bacterial genus Staphylococcus comprises a large group of pathogenic and nonpathogenic species associated with an array of host species. Staphylococci are differentiated into coagulase-positive or coagulase-negative groups based on the capacity to promote clotting of plasma, a phenotype historically associated with the ability to cause disease. However, the genetic basis of this important diagnostic and pathogenic trait across the genus has not been examined to date. Here, we selected 54 representative staphylococcal species and subspecies to examine coagulation of plasma derived from six representative host species. In total, 13 staphylococcal species mediated coagulation of plasma from at least one host species including one previously identified as coagulase negative (Staphylococcus condimenti). Comparative genomic analysis revealed that coagulase activity correlated with the presence of a gene (*vwb*) encoding the von Willebrand binding protein (vWbp) whereas only the Staphylococcus aureus complex contained a gene encoding staphylocoagulase (Coa), the classical mediator of coagulation. Importantly, S. aureus retained *vwb*-dependent coagulase activity in an S. aureus strain deleted for *coa* whereas deletion of *vwb* in Staphylococcus pseudintermedius resulted in loss of coagulase activity. Whole-genome-based phylogenetic reconstruction of the Staphylococcus genus revealed that the *vwb* gene has been acquired on at least four different occasions during the evolution of the Staphylococcus genus followed by allelic diversification via mutation and recombination. Allelic variants of vWbp from selected coagulase-positive staphylococci mediated coagulation in a host-dependent manner indicative of host-adaptive evolution. Taken together, we have determined the genetic and evolutionary basis of staphylococcal coagulation, revealing vWbp to be its archetypal determinant.

**IMPORTANCE** The ability of some species of staphylococci to promote coagulation of plasma is a key pathogenic and diagnostic trait. Here, we provide a comprehensive analysis of the coagulase positivity of the staphylococci and its evolutionary genetic basis. We demonstrate that the von Willebrand binding protein rather than staphylocoagulase is the archetypal coagulation factor of the staphylococci and that the *vwb* gene has been acquired several times independently during the evolution of the staphylococci. Subsequently, *vwb* has undergone adaptive diversification to facilitate host-specific functionality. Our findings provide important insights into the evolution of pathogenicity among the staphylococci and the genetic basis for a defining diagnostic phenotype.

## INTRODUCTION

The Staphylococcus genus comprises at least 70 species and subspecies associated with an array of host species and habitats (1). The defining phenotype for the identification of the major human and animal pathogen Staphylococcus aureus has traditionally been coagulation of rabbit plasma. However, in addition to S. aureus there are eight described coagulase-positive staphylococci (CoPS) including Staphylococcus argenteus, Staphylococcus cornubiensis, Staphylococcus delphini, Staphylococcus intermedius, Staphylococcus lutrae, Staphylococcus pseudintermedius, Staphylococcus coagulans, and Staphylococcus schweitzeri, with Staphylococcus agnetis, Staphylococcus chromogenes, and Staphylococcus hyicus described as coagulase variable (2, 3). To date, the basis for the coagulation phenotype has been examined only for S. aureus with staphylocoagulase (Coa) considered to be the classical mediator of coagulation. However, the von Willebrand binding protein (vWbp) also exhibits coagulase activity (4, 5). Of note, a staphylococcal pathogenicity island (SaPI)-encoded vWbp associated with ruminant or equine strains of S. aureus confers the ability to coagulate plasma from their cognate host species, demonstrating a role for SaPI-encoded vWbp in host adaptation (6, 7).

**FIG 1:**
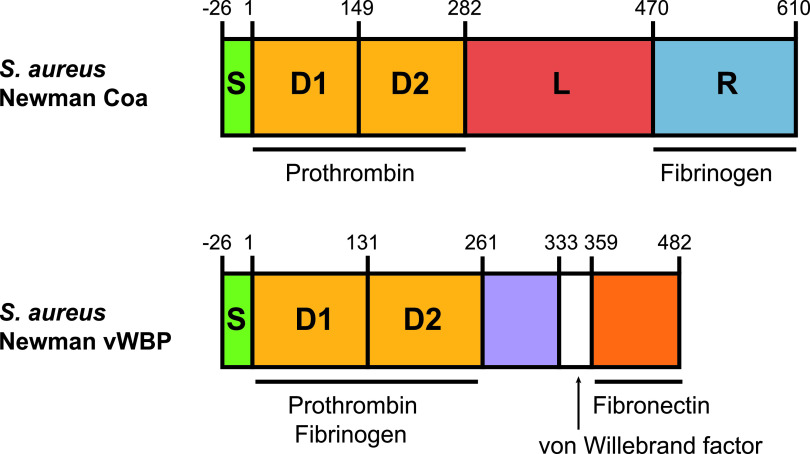
Schematic protein domain structure of Coa and vWbp of S. aureus Newman. Both Coa and vWbp contain N-terminal signal peptides (S) and prothrombin-binding D1 and D2 domains. The C-terminal sequence of Coa contains a linker (L) followed by fibrinogen-binding repeats (R). The C-terminal sequence of vWbp contains a von Willebrand factor binding region between two domains. In S. aureus Newman, Coa and vWbp share 26.3% amino acid identity.

For both S. aureus Coa and vWbp, which contain 26.3% amino acid identity in strain Newman, the N-terminal D1 and D2 domains are sufficient for the coagulation of plasma via the nonproteolytic activation of prothrombin, which in turn cleaves fibrinogen to form insoluble fibrin, thus promoting clot formation (Fig. 1) (8, 9). Allelic variation of the D1 and D2 domains in both Coa and vWbp is associated with attenuated binding efficiencies to prothrombin from different host species, underpinning the host species-dependent coagulation observed (5, 10–12). For S. aureus, mediators of coagulation promote bacterial survival in fibrin clots, allowing persistence in a murine subcutaneous infection model and bacterial dissemination in a murine bloodstream infection model (13–15). Both *coa* and *vwb* genes are also required for the development of kidney microabscesses during invasive disease (14, 16, 17). The key role of both Coa and vWbp in the pathogenesis of S. aureus disease suggests that the capacity for coagulation of plasma by other staphylococci is likely to be relevant to pathogenicity (8). A recent study used PCR to identify genes more similar to *vwb* than *coa* among four non-*aureus* CoPS (18). Furthermore, a recombinant form of a protein encoded by S. pseudintermedius orthologous to vWbp of S. aureus was demonstrated to have coagulase activity (19). However, the evolutionary genetic and functional basis of the coagulase phenotype for non-*aureus* staphylococci has not been examined to date.

Here, we perform a comprehensive analysis of the coagulation capacity of 54 representative staphylococcal species and subspecies in plasma from multiple host species. Combining comparative genomic, evolutionary, and functional analyses, we examined the molecular basis for coagulation among CoPS. Importantly, we demonstrate that vWbp is the archetypal coagulation factor of CoPS, whereas the classical Coa is limited to members of the S. aureus complex only. Acquisition of the *vwb* gene occurred on four independent occasions during the evolutionary history of the staphylococci and was followed by diversification via recombination and host-specific functional specialization. Taken together, these findings reveal the evolutionary history of a key diagnostic and pathogenic trait of staphylococci.

## RESULTS

### Comprehensive examination of CoPS and host species-associated coagulation.

Of the 71 known staphylococcal species and subspecies that comprise the Staphylococcus genus (1), 12 are reported to mediate coagulation of rabbit plasma (2). However, our previous findings indicate host-dependent variation in susceptibility to plasma coagulation (6, 7). Here, we investigated the capacity for 51 representative staphylococcal species and 3 subspecies to mediate coagulation of plasma from six host species including human, canine, equine, porcine, avian, and rabbit (see Table S1 in the supplemental material). We also included five *Mammaliicoccus* species previously classified as members of the family *Staphylococcaceae* (20). A coagulation phenotype was observed for 13 staphylococcal species in plasma from at least one host species (Table 1). Unexpectedly, Staphylococcus condimenti, previously determined to be coagulase negative (21), exhibited coagulation of equine plasma. Considerable variation was observed in the capacity for different staphylococcal species to coagulate plasma from different host species, with coagulation of human plasma restricted to S. aureus, *S. chromogenes*, and *S. schweitzeri* (Table 1). Of note, *S. condimenti* and *S. hyicus* did not coagulate rabbit plasma (traditionally used for identifying the coagulase phenotype in clinical diagnostic labs) but mediated coagulation of plasma from other host species. Furthermore, the time to coagulation mediated by CoPS varied from 2 to 24 h according to host species (Table 1). In summary, this genus-wide analysis of coagulation using plasma from multiple host species reveals considerable variation in the efficiency of plasma coagulation depending on the host species and suggests a revision of the group of CoPS to include *S. condimenti*.

**TABLE 1:** Coagulase activity of staphylococci in plasma from six host species

Species	Associated host	Coagulase activity in plasma type^*a*^:					
		Human	Canine	Equine	Porcine	Avian	Rabbit
*S. agnetis*	Bovine	−	+	+	+++	−	+
*S. argenteus*	Human	−	+++	++	++	−	++++
S. aureus	Human	++++	+	+++	++	−	++++
*S. chromogenes*	Bovine	+++	+++	−	−	−	+++
*S. coagulans*	Canine	−	++	++	−	++	+++
*S. condimenti*	Unknown	−	−	+	−	−	−
*S. cornubiensis*	Human	−	+++	++++	++++	+++	++++
S. delphini	Dolphin	−	+++	++	+++	+	+++
*S. hyicus*	Porcine	−	++	−	++++	−	−
S. intermedius	Avian	−	+++	−	−	−	++++
*S. lutrae*	Otter	−	+++	++	−	+	++++
S. pseudintermedius	Feline	−	+++	+++	++	−	+++
*S. schweitzeri*	Monkey	+	+	+	−	−	+

a Only species that demonstrated coagulation of any plasma type in all three biological replicates are shown. +, ++, +++, and ++++, coagulating activity observed after 24, 6, 4, or 2 h of incubation, respectively; −, no coagulating activity detected after 24 h.

10.1128/mSphere.00381-21.4 TABLE S1 Staphylococcus and *Mammaliicoccus* species tested for coagulation. Download Table S1, DOCX file, 0.01 MB. Copyright © 2021 Pickering et al. 2021 Pickering et al. https://creativecommons.org/licenses/by/4.0/ This content is distributed under the terms of the Creative Commons Attribution 4.0 International license.

### The capacity for coagulase activity was acquired on multiple occasions during the evolution of the staphylococci.

**FIG 2:**
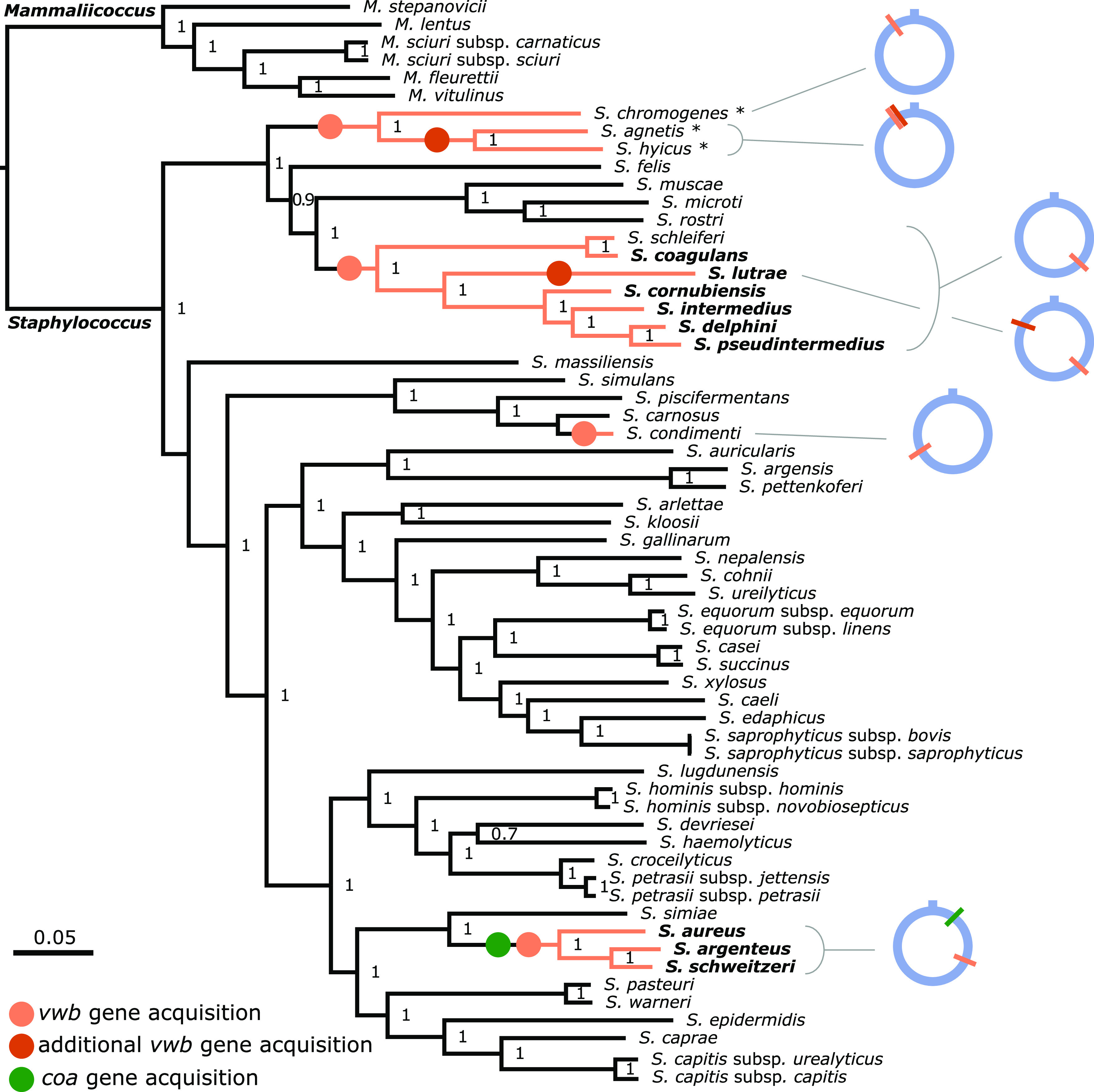
All CoPS species contain the *vwb* gene. Maximum likelihood phylogenetic tree of 50 staphylococcal species and 5 additional subspecies based on a core genome alignment of 231 concatenated genes. A list of the genome sequences used is included in Table S2. Coagulase-positive species are highlighted in bold, and coagulase-variable species are highlighted with an asterisk. Species containing the *vwb* gene are represented with orange branches, with orange circles indicating the branch on which this gene was acquired. Red circles indicate the nodes in which additional *vwb* copies were acquired. The single acquisition event of the *coa* gene by an ancestor of the S. aureus complex is indicated by a green circle. Genome locations of the *vwb* and *coa* genes in each CoPS lineage are represented by colored bars on a blue ring depicting the chromosome. Support values are shown on each branch and were calculated using the Shimodaira-Hasegawa test. The *Mammaliicoccus* cluster is the root of the tree.

To examine the distribution of CoPS species across the staphylococcal phylogeny, we constructed the most comprehensive species phylogeny to date based on 231 concatenated core genes present in 50 staphylococcal species (five of which included two subspecies each) for which the whole-genome sequences were available (Table S2). CoPS are distributed into three distinct clades including the S. aureus complex (S. aureus, *S. argenteus*, and *S. schweitzeri* [22]), the Hyicus group containing coagulase-variable species (*S. hyicus*, *S. agnetis*, and *S. chromogenes* [3, 23, 24]), and the Intermedius group (S. intermedius, S. delphini, S. pseudintermedius, *S. cornubiensis*, Staphylococcus ursi, *S. lutrae*, S. schleiferi, and *S. coagulans* [25–28]), as previously described (Fig. 2) (28–30).

10.1128/mSphere.00381-21.5 TABLE S2 Genome sequences used in this study. Download Table S2, DOCX file, 0.01 MB. Copyright © 2021 Pickering et al. 2021 Pickering et al. https://creativecommons.org/licenses/by/4.0/ This content is distributed under the terms of the Creative Commons Attribution 4.0 International license.

To investigate the genetic basis for the coagulase-positive phenotype, we examined the genus-wide distribution of genes known to encode proteins with coagulase activity (Fig. 2). Unexpectedly, we found that the *coa* gene (encoding the classical staphylocoagulase) was restricted to the S. aureus complex, but the *vwb* gene was identified among all CoPS in addition to coagulase-negative staphylococcus (CoNS) *S. schleiferi* (Fig. 2). The *vwb* genes were identified at distinct genomic locations in each coagulase-positive clade, suggesting that acquisition occurred independently via horizontal transfer on at least four occasions (Fig. 2). *S. agnetis* and *S. hyicus* each have two *vwb* copies in tandem, presumably as a result of a gene duplication event that occurred prior to divergence of these two closely related species. *S. lutrae* also carries two copies of *vwb*, one in the same genomic location as the other members of the Hyicus clade and the second copy in a unique genomic location (Fig. 2). Taken together, we infer that *vwb* was acquired on multiple occasions during the evolutionary history of the staphylococci, leading to the emergence of distinct groups of CoPS.

The presence of the *vwb* gene in *S. condimenti* (and the lack of a *vwb* gene in the closely related species Staphylococcus carnosus) indicates an independent acquisition during the evolution of this species, which correlates with the capacity to coagulate equine plasma as identified in the current study (Table 1). The presence of the *vwb* gene in both the Hyicus and Intermedius coagulase-positive clades, and the absence of *coa*, suggested that vWbp may be the mediator of coagulation for these species.

### vWbp diversification has occurred via mutation, recombination, and duplication.

**FIG 3:**
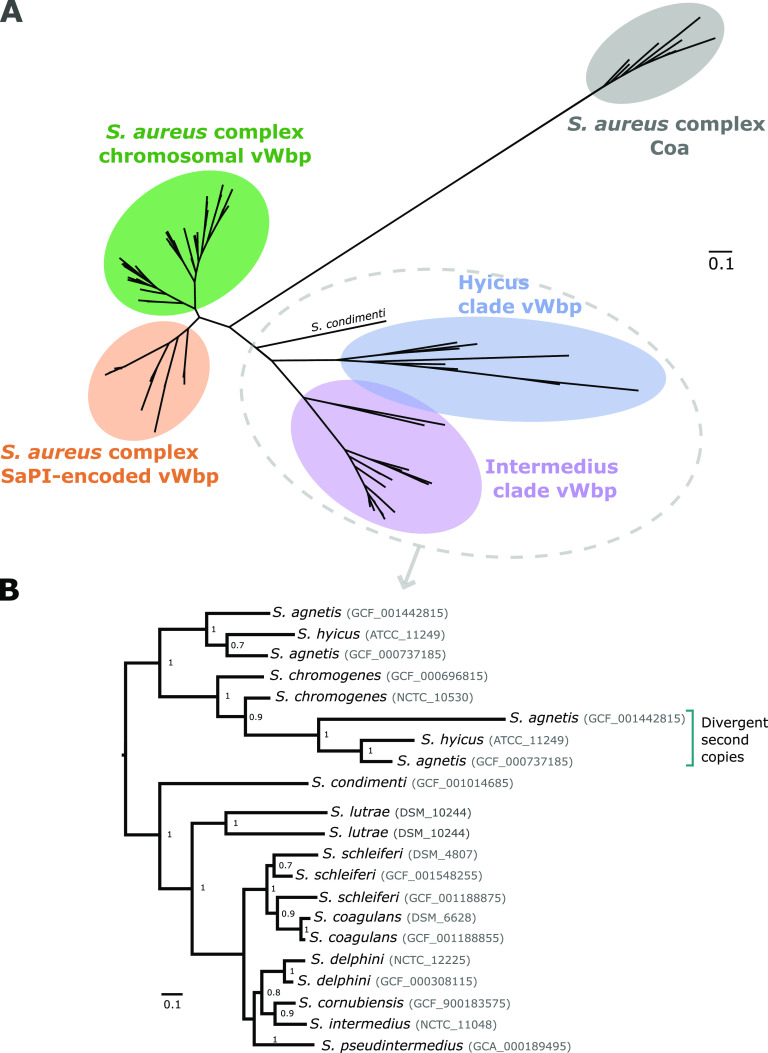
Staphylococcal vWbp has an evolutionary history distinct from that of coagulase. (A) Unrooted maximum-likelihood tree constructed using FastTree v2 of the Coa and vWbp protein sequences. Each of the main clusters is highlighted and labeled. Coagulase positivity has evolved on multiple occasions through acquisition of *vwb* followed by evolution according to species. (B) Phylogenetic reconstruction of the non-*aureus* CoPS based on vWbp protein sequences.

To investigate the diversity and relatedness of the *vwb* and *coa* genes among the CoPS, a phylogenetic tree was constructed based on sequences extracted from genomes representing 41 non-S. aureus species (Table S2), and 802 S. aureus, *S. schweitzeri*, and *S. argenteus* genomes (31). From those, we selected representative copies of each phylogenetic cluster within the S. aureus complex to avoid redundancy, leaving a total of 12 Coa and 129 vWbp protein sequences from 11 species across the three CoPS clades (Fig. 3). vWbp sequences form four distinct clusters that correspond to chromosomal genes from the Hyicus (*n* = 8) and Intermedius (*n* = 32) clades, the S. aureus complex (*n* = 51), and SaPI-associated genes from S. aureus (*n* = 37), respectively. The only vWbp copy from the only *S. condimenti* genome was most closely related to the Hyicus clade vWbp copies (Fig. 3). The vWbp clades are more closely related to each other (34.7% average pairwise protein similarity between the full-length vWbp alleles of the Hyicus and Intermedius clades) than to the S. aureus Coa protein (22.6% and 21.6% average pairwise protein similarity between the full-length Coa and vWbp alleles of the Hyicus and Intermedius clades, respectively), which forms a monophyletic clade indicating a distinct evolutionary history (Fig. 3; also see Table S3). The topology of the vWbp Hyicus and Intermedius clades correlates with the species distribution in the core genome-based Staphylococcus genus phylogeny (Fig. 2), consistent with ancient independent acquisition by progenitors of the Hyicus and Intermedius clades, followed by evolution according to species and a low degree of interspecies gene exchange (Fig. 3). The exception to this were the paralogous *vwb* genes in *S. agnetis* and *S. hyicus*, which have distinct evolutionary histories compared to the ancestral chromosomal copy, whereas the *S. lutrae vwb* copies clustered together in the phylogeny, consistent with gene duplication since speciation (Fig. 3).

10.1128/mSphere.00381-21.6 TABLE S3 Average pairwise protein similarity between full-length vWbp and Coa sequences or the D1 and D2 prothrombin-binding domains. Download Table S3, DOCX file, 0.01 MB. Copyright © 2021 Pickering et al. 2021 Pickering et al. https://creativecommons.org/licenses/by/4.0/ This content is distributed under the terms of the Creative Commons Attribution 4.0 International license.

**FIG 4:**
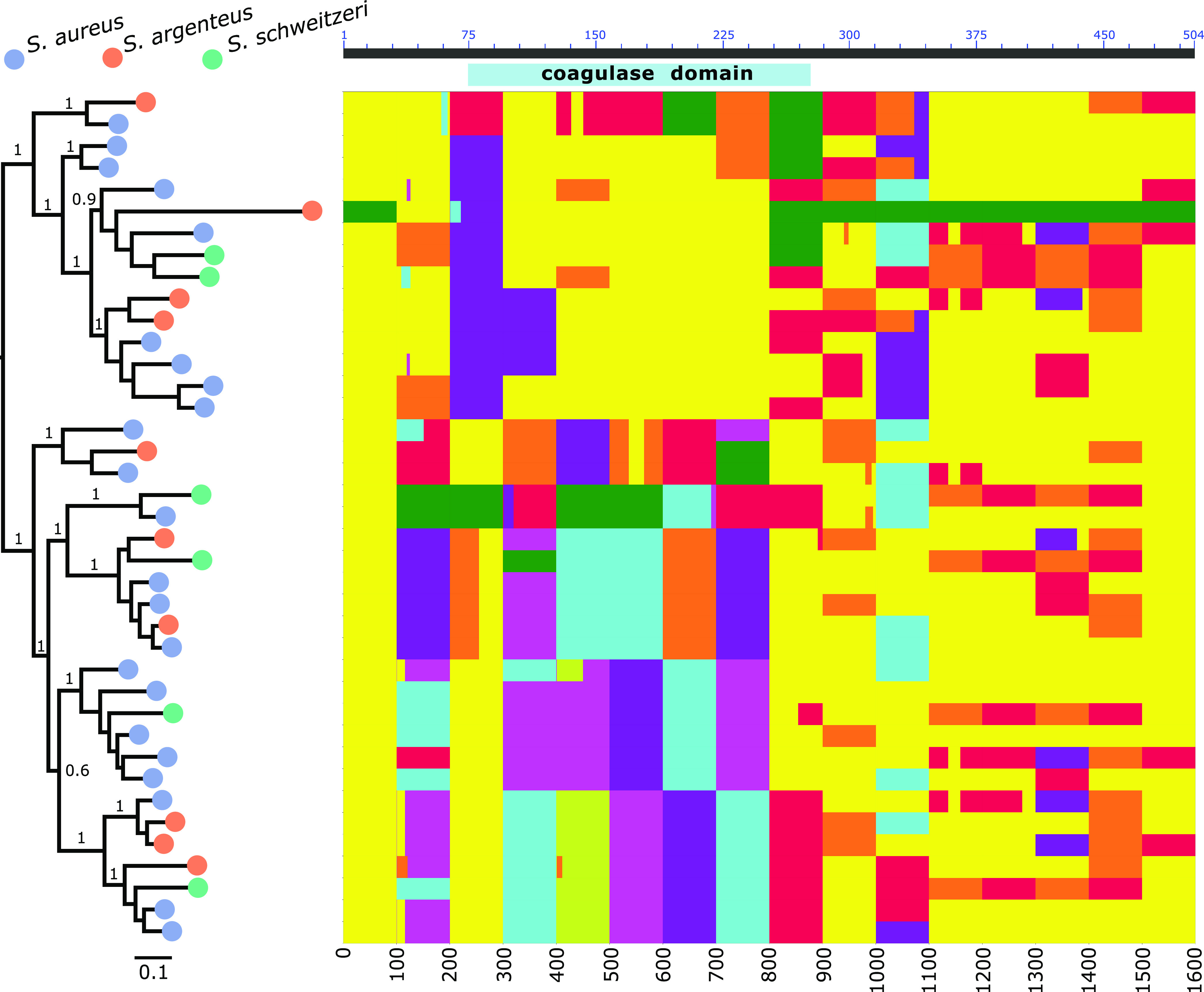
The chromosomal *vwb* has undergone extensive recombination among the S. aureus complex species. Midpoint-rooted maximum-likelihood tree constructed using FastTree v2 of the S. aureus complex *vwb* nucleotide sequences. Colored circles denote each Staphylococcus species. Plot of the FastGEAR analysis of the *vwb* nucleotide alignment applied to 100-bp-long windows. For each window (i.e., column in the plot), each color represents different phylogenetic clusters based on the BAPS algorithm. The axis at the top shows, for reference, the location of the coagulase (D1+D2) domain using the amino acid coordinates of the RF122 S. aureus isolate vWbp.

Previously, it was shown that recombination has contributed to the evolution of the *coa* genes among S. aureus isolates (12). In order to examine the impact of recombination on the evolutionary history of the *vwb* gene in the S. aureus complex, we employed both RDP4 and FastGEAR analysis using a 100-bp sliding window approach on 39 *vwb* gene sequences representing the breadth of sequence diversity. This analysis revealed high levels of recombination affecting the N-terminal D1 and D2 coagulation domains, explaining the lower pairwise protein similarity observed in the S. aureus complex for the D1 and D2 domains, in comparison to full-length vWbp (Fig. 4; Table S3). Of note, S. aureus complex chromosomal *vwb* copies exhibited extensive sequence admixture between S. aureus, *S. argenteus*, and *S. schweitzeri* consistent with a shared ecology providing opportunities for horizontal gene transfer and recombination (Fig. 4).

Taken together, we identified a complex evolutionary history of the *vwb* gene with evidence for diversification of *vwb* in a species-dependent manner in the Hyicus and Intermedius clades. In contrast, a high degree of recombination between the members of the S. aureus complex has generated extensive allelic diversity of *vwb*.

### vWbp is the archetypal coagulation factor of the staphylococci.

**FIG 5:**
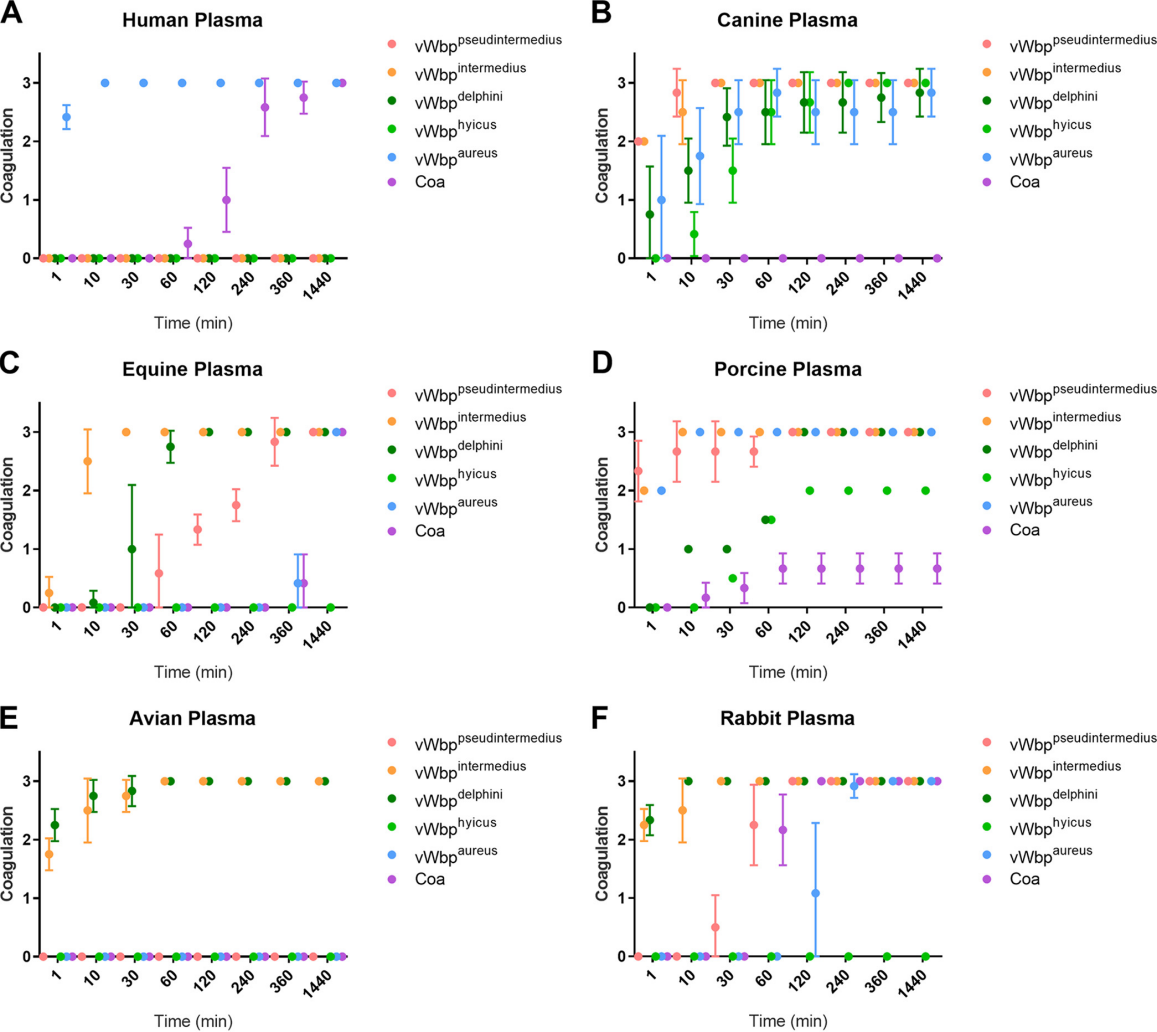
Recombinant vWbp proteins exhibit host-specific coagulation of plasma. Coagulation of plasma from human (A), canine (B), equine (C), porcine (D), avian (E), and rabbit (F) origin by 50 μg ml^−1^ recombinant vWbp encoded by S. pseudintermedius (pink), S. intermedius (orange), S. delphini (dark green), *S. hyicus* (light green), and S. aureus (blue), in addition to S. aureus Coa (purple). Coagulation was scored as 0.5 for trace levels of coagulation, 1.0 to 2.5 for partially coagulating plasma, and 3.0 for a complete clot. Data are shown as the mean from 6 replicates with error bars representing standard deviation.

From our phylogenetic analysis of the distribution of genes encoding coagulase activity, and the correlation of the *vwb* gene with coagulase positivity, we hypothesized that *vwb* is the main mediator of coagulation in the Staphylococcus genus. To date, the capacity for vWbp from non-*aureus* CoPS to mediate coagulation has not been tested. Accordingly, we produced recombinant vWbp variants derived from CoPS species including human S. aureus Newman, canine S. pseudintermedius ED99, avian S. intermedius ATCC 29663, equine S. delphini 8086, and porcine *S. hyicus* ATCC 11249 in addition to Coa from S. aureus Newman and tested their capacity to promote coagulation of plasma from an array of different host species in dose-dependent assays (Fig. 5; Table 2; Fig. S1 to S3) (23, 32–35). Each variant had the capacity to mediate plasma coagulation from their cognate host species, though there was considerable variation in the capacity to coagulate plasma from other species (Fig. 5; Table 2). Overall, the activity was similar to that observed for the parent bacterial species (Table 1), consistent with vWbp being the key mediator of coagulation in non-*aureus* staphylococci.

**TABLE 2:** Minimal concentration of recombinant vWbp or Coa protein required to coagulate plasma after 24 h

Protein	Minimal concn (μg ml^−1^) for plasma type^*a*^:					
	Human	Canine	Equine	Porcine	Avian	Rabbit
Coa^aureus^	25		50			25
vWbp^aureus^	25	25	50	25		25
vWbp^intermedius^		25	5	25	25	5
vWbp^pseudintermedius^		25	25	50		25
vWbp^delphini^		5	5	25	25	5
vWbp^hyicus^		25		50		

a Values are the minimal concentration of recombinant protein required to induce coagulation after 24 h. Recombinant protein was applied to PBS-diluted plasma at 50, 25, 5, or 1 μg ml^−1^.

10.1128/mSphere.00381-21.1 FIG S1 Recombinant vWbp proteins at 50 μg ml^−1^ exhibit different host-specific coagulation phenotypes. Download FIG S1, DOCX file, 0.6 MB. Copyright © 2021 Pickering et al. 2021 Pickering et al. https://creativecommons.org/licenses/by/4.0/ This content is distributed under the terms of the Creative Commons Attribution 4.0 International license.

**FIG 6:**
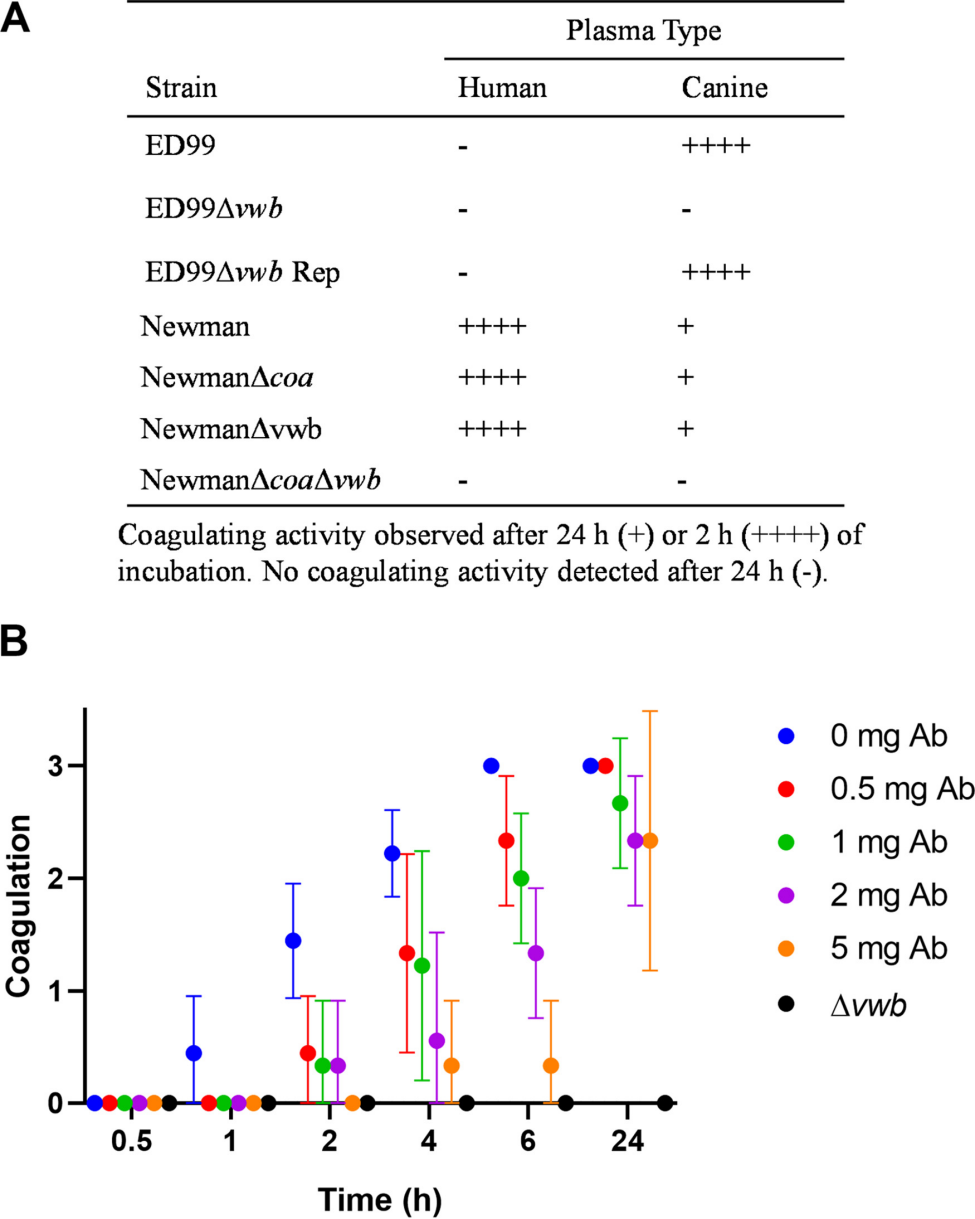
*vwb* is required for the coagulase phenotype of S. pseudintermedius ED99. (A) Coagulation assay of wild-type and mutant strains of S. pseudintermedius ED99 and S. aureus Newman in human and canine plasma. Average data are provided from three biological replicates. (B) Antibody (Ab)-mediated inhibition of coagulation in canine plasma using ED99 wild type in the presence of increasing concentrations of anti-vWbp^pseudintermedius^ antibody. The mean values are given from three experiments with error bars representing standard deviation (*n* = 3).

To confirm this in a representative CoPS species of clinical relevance, we constructed a *vwb* deletion mutant in S. pseudintermedius ED99 (34), reintroduced the *vwb* gene to produce a repaired derivative strain, and also employed single and double mutants of *vwb* and *coa* in S. aureus strain Newman (16). Single deletions of the *vwb* and *coa* genes in S. aureus Newman did not ablate plasma coagulation, with deletion of both genes required for loss of the coagulation phenotype (Fig. 6A). In contrast, deletion of *vwb* in S. pseudintermedius (ED99Δ*vwb*) resulted in loss of ability to coagulate plasma after 24 h whereas the isogenic wild-type and ED99Δ*vwb* repaired strain produced complete clots after 2 h (Fig. 6A). Furthermore, antibodies specific for vWbp^pseudintermedius^ delayed coagulation in a dose-dependent manner, confirming that vWbp mediates plasma coagulation by S. pseudintermedius ED99 (Fig. 6B). These data demonstrate that vWbp is required for the coagulase positivity of S. pseudintermedius ED99.

Taken together, these evolutionary and functional data support the hypothesis that vWbp is the major determinant of coagulase positivity among staphylococci. For the S. aureus complex species only, Coa provides redundancy in coagulase activity. In addition, allelic diversification has resulted in host-adaptive evolution supporting the capacity of CoPS to adapt to different host niches.

## DISCUSSION

The ability to coagulate plasma is considered a defining phenotype for the identification of the major human pathogen S. aureus. Furthermore, the importance of coagulases Coa and vWbp in S. aureus pathogenesis is well established for catheter-related biofilm formation and bacterial survival during bloodstream infections (13, 15, 36, 37). However, the evolutionary and genetic basis of coagulation for non-*aureus* staphylococci or its role in pathogenesis had not been dissected previously. Here, we have performed a comprehensive phylogenetic and functional analysis of coagulase positivity among the staphylococci. The ability to coagulate plasma has evolved on multiple occasions across the genus via independent acquisitions of the *vwb* gene whereas the *coa* gene was likely acquired on a single occasion by an ancestor of the S. aureus complex (Fig. 2). Our functional analyses demonstrated that the *vwb* gene identified among CoPS mediates coagulation of plasma in a host-dependent manner and is solely responsible for the coagulase phenotype in a representative non-*aureus* species, S. pseudintermedius (Fig. 5 and 6). Taken together, these data suggest that vWbp is the archetypal coagulation factor of the Staphylococcus genus.

We identified extensive allelic diversity and recombination of the *vwb* gene among members of the S. aureus complex (Fig. 3), consistent with an overlapping habitat for S. aureus, *S. argenteus*, and *S. schweitzeri* (Fig. 4). A previous study has demonstrated gene flow between S. aureus and *S. argenteus* via recombination, driven by a possible shared ecological niche in livestock (38). In spite of the evidence for horizontal transfer of *vwb* sequences, SaPI-encoded variants of vWbp were restricted to S. aureus, which may reflect its multihost ecology and the role of vWbp in host adaptation (Fig. 3) (6). However, whole-genome sequencing of other species is very limited compared to S. aureus, and expanded sequencing of *S. schweitzeri* and *S. argenteus* isolates may reveal novel variants. Of note, extensive variation and evidence for recombination have previously been reported for the *coa* gene of S. aureus and *S. argenteus* (12, 39, 40). In contrast, our *vwb* phylogenetic analysis for non-*aureus* staphylococci supported a model of initial *vwb* gene acquisition followed by diversification according to species via mutation rather than interspecies recombination (Fig. 3).

In order to examine the possibility that the allelic variation of *vwb* between staphylococcal species in part reflected the independent evolution within distinct host ecologies, we examined the coagulase activity of recombinant vWbp from representative CoPS species in plasma from six different host species. In each case, recombinant vWbp variants exhibited coagulation of plasma from their cognate host species and various levels of coagulation from other host species (Table 2; Fig. 5). The host species-dependent activity of vWbp presumably reflects variation in the sequence of prothrombin, the main substrate for vWbp (10, 11). Overall, these data indicate that *vwb* has undergone adaptive evolution to facilitate functional activity in its preferred host species, highlighting an important role for vWbp in host-pathogen interactions.

The coagulase test has been used since the 1940s to distinguish the virulent S. aureus from less-virulent CoNS (41). Since the 1970s, additional CoPS have been identified, largely associated with infections of animals (33), and the coagulase test has been widely used in human and veterinary medicine. Although the coagulase test is still commonly used, particularly in resource-poor settings around the world, molecular genetic or proteomic approaches have largely supplanted biochemical testing (42). Our findings suggest that *vwb* sequence analysis to identify alleles associated with specific CoPS would offer a robust and simple diagnostic approach for the identification of CoPS to species level and should be performed when describing novel staphylococcal species. Of 14 species that contained a *vwb* gene, *S. schleiferi* was the only one which did not exhibit a coagulase phenotype. This may reflect lack of gene expression or a distinct host tropism that was not represented among the plasma samples included, and analysis of additional isolates would be required to address this. Similarly, an understanding of the genetic basis for the variation in coagulase positivity among some staphylococcal species would require genomic analysis of more isolates. Of note, we examined the genome sequences of the newly identified Staphylococcus durrellii, Staphylococcus lloydii, and Staphylococcus pseudoxylosus type strains and did not identify a *vwb*-like gene, consistent with their characterization as CoNS (43, 44). Furthermore, the genome of the recently identified Staphylococcus ursi type strain MI 10-1553^T^, reported as a CoNS of the otherwise coagulase-positive Intermedius clade, contains a *vwb*-like remnant consistent with a deletion event that abrogated the ability to coagulate plasma (26).

In conclusion, the current study provides the first comprehensive genetic and functional analysis of the coagulation capacity of the Staphylococcus genus. We propose that vWbp, rather than Coa, is the archetypal coagulation factor of CoPS across the genus with the *vwb* gene acquired independently on multiple occasions, leading to distinct coagulase-positive clades within the Staphylococcus phylogeny. Diversification of the *vwb* gene has occurred via mutation or recombination generating allelic diversity, which in part reflects adaptive evolution to enable functionality of vWbp in the preferred host species, suggesting a key role in host-pathogen interactions. Taken together, our findings provide clarity into the evolutionary genetic and functional basis for a defining diagnostic and pathogenic phenotype of the Staphylococcus genus.

## MATERIALS AND METHODS

### Phylogenetic analysis.

Whole-genome sequences of the staphylococcal and mammaliicoccal type strains were obtained from NCBI (see Table S2 in the supplemental material). Each genome was annotated with Prokka v1.12 (45), and a core genome alignment was generated using Roary v3.12 (46), based on a minimum percent identity cutoff of 80. The core genome alignment was then used to build a maximum likelihood phylogenetic tree with FastTree v2.1.7 (47). Coagulase and vWbp amino acid sequences were extracted using Protein BLAST (blastp) from the reference whole-genome sequences from a genome sequence data set representative of the global S. aureus diversity (31). Sequences were aligned using the ProbCons algorithm (48) implemented in Jalview v2 (49). Phylogenetic trees were constructed using FastTree, and recombination analysis was performed using the programs Recombination Detection Program (RDP4) (50) and FastGEAR (51). Average pairwise protein similarities were calculated using MEGA X v10.1.8 (52) after classifying the D1 and D2 domains using the Conserved Domain Database (53).

### Coagulation assay.

The bacterial strains used for coagulation assays are detailed in Table S1. All strains were cultured overnight in brain heart infusion (BHI) broth at 37°C with shaking. For bacterial coagulation assays, the cells were diluted in phosphate-buffered saline (PBS) to an optical density at 600 nm (OD_600_) of 1.0. For recombinant protein coagulation assays, each recombinant protein was diluted in PBS to the required concentration using the Nanodrop ND-1000 spectrophotometer. Plasma from all hosts was sourced from SeraLab, Lampire, or BioIVT in sodium citrate and diluted 1:3 in PBS before use. Coagulation was tested by mixing 190 μl of diluted plasma with 10 μl of recombinant protein or bacterial cells in sterile borosilicate glass tubes. All coagulation assays were performed statically at 37°C in biological triplicate with the level of coagulation observed at 1, 2, 4, 6, and 24 h by tilting the tubes. Antibody blocking of coagulation was performed using IgY antibody generated against vWbp^pseudintermedius^ after lipopolysaccharide (LPS) removal (Gallus Immunotech) at a concentration of 5, 2, 1, 0.5, or 0.1 mg in the 200-μl reaction mixture with concentrated supernatant.

### Allelic replacement of S. pseudintermedius ED99.

Mutant and repaired strains of S. pseudintermedius ED99 were generated using the allele replacement plasmid pIMAY and the primers detailed in Table S4, as described previously (54, 55). Briefly, the upstream and downstream flanking regions of the *vwb* gene were amplified by PCR and annealed by splicing PCR to generate a deleted form of the gene. For the repaired mutant, primers were designed to amplify the full-length *vwb* gene in addition to upstream and downstream flanking regions with a synonymous mutation introduced into the *vwb* gene to allow identification of the repaired strain in comparison to the wild type. Restriction digestion with EcoRI (NEB) and T4 ligation (NEB) into pIMAY was carried out prior to transformation of the pIMAY::*vwb* and pIMAY::*vwb* Rep constructs into Escherichia coli DC10B cells, followed by transformation into S. pseudintermedius ED99 or ED99Δ*vwb*. Allele replacement was performed as previously described (55), and genetically manipulated strains were validated by whole-genome sequencing, growth analysis in BHI using a FLUOstar Optima microplate reader (600 nm), and Western blot analysis of vWbp expression using 5 μg ml^−1^ anti-vWbp^pseudintermedius^ IgY and 0.5 μg ml^−1^ F(ab′)_2_ rabbit anti-chicken IgG-horseradish peroxidase (HRP) (Bethyl Laboratories).

10.1128/mSphere.00381-21.7 TABLE S4 Primers used in this study. Download Table S4, DOCX file, 0.01 MB. Copyright © 2021 Pickering et al. 2021 Pickering et al. https://creativecommons.org/licenses/by/4.0/ This content is distributed under the terms of the Creative Commons Attribution 4.0 International license.

### Expression and purification of recombinant proteins.

The N-terminal amino acids are important for the function of coagulase, with N-terminal modification of vWbp impairing coagulation efficiency (56). In order to use the C-terminal His tag expression construct pET21b with the NheI site, all additional plasmid N-terminal amino acids (Met-Ala-Ser-Met) were removed from the plasmid using pET21b-specific primers (Table S4). This modified plasmid was used to clone *vwb*^hyicus^, *vwb*^aureus^, and *coa*^aureus^. All pET21b constructs were produced using standard restriction digestion and NheI, NdeI, and XhoI (NEB) and T4 ligase (NEB) procedures. Expression constructs were expressed in BL21(DE3) E. coli cells (NEB) using 1 mM isopropyl-β-d-thiogalactopyranoside (IPTG) for 4 h at 37°C. Cell lysates were produced at 20 kpsi using a One Shot cell disruptor (Constant Systems). All recombinant proteins underwent two purification steps using the high-performance liquid chromatography (HPLC) Äkta system (GE Healthcare): first, immobilized metal affinity chromatography with 1 ml Ni-nitrilotriacetic acid (NTA) superflow columns (Qiagen) in either native or denaturing purification buffers, and second, ion-exchange chromatography using Q Sepharose (GE Healthcare) for positively charged proteins or SP Sepharose (GE Healthcare) for negatively charged proteins. All proteins were dialyzed to PBS using Float-A-Lyzers (Spectrum Laboratories) and analyzed by SDS-PAGE.

10.1128/mSphere.00381-21.2 FIG S2 Recombinant vWbp proteins at 25 μg ml^−1^ exhibit different host-specific coagulation phenotypes. Download FIG S2, DOCX file, 0.6 MB. Copyright © 2021 Pickering et al. 2021 Pickering et al. https://creativecommons.org/licenses/by/4.0/ This content is distributed under the terms of the Creative Commons Attribution 4.0 International license.

10.1128/mSphere.00381-21.3 FIG S3 Recombinant vWbp proteins at 5 μg ml^−1^ exhibit different host-specific coagulation phenotypes. Download FIG S3, DOCX file, 0.6 MB. Copyright © 2021 Pickering et al. 2021 Pickering et al. https://creativecommons.org/licenses/by/4.0/ This content is distributed under the terms of the Creative Commons Attribution 4.0 International license.
