# Supplementary material for: Evolutionary and Functional Analysis of Coagulase Positivity among the Staphylococci
Source: mSphere. 2021 Aug 4;6(4):e00381-21. doi: 10.1128/mSphere.00381-21 (PMC8386474; doi:10.1128/mSphere.00381-21)
Supplement: TABLE S1 [file msphere.00381-21-st001.docx]

TABLE S1. *Staphylococcus* and *Mammaliicoccus* species tested for coagulation

| Species | Strain | Host Origin |
| --- | --- | --- |
| *M. fleuretti* | DSM 13212 | Goat milk |
| *M. lentus* | ATCC 29070 | Goat udder |
| *M. sciuri* subsp. *sciuri* | DSM 20345 | Squirrel skin |
| *M. stepanovicii* | DSM 26319 | Bank vole |
| *M. vitulinus* | DSM 15615 | Ground lamb |
| *S. agnetis* | DSM 23656 | Cow mastitic milk |
| *S. argensis* | DSM 29875 | River sediment |
| *S. argenteus* | DSM 28299 | Human blood culture |
| *S. arlettae* | ATCC 43957 | Poultry skin |
| *S. aureus subsp. aureus* | Newman | Human |
| *S. aureus subsp. anaerobius* | ATCC 35844 | Sheep abscess |
| *S. auricularis* | ATCC 33753 | Human Ear |
| *S. caeli* | NCTC 14063 | Air of rabbit holding |
| *S. capitis* subsp. *capitis* | ATCC 27840 | Human skin |
| *S. capitis* subsp. *urealyticus* | ATCC 49324 | Human ear |
| *S. caprae* | ATCC 35538 | Goat milk |
| *S. carnosus* subsp*. carnosus* | ATCC 51365 | Sausage |
| *S. chromogenes* | DSM 20454 | Pig |
| *S. coagulans* | DSM 6628 | Dog ear |
| *S. cohnii* | ATCC 29974 | Human skin |
| *S. condimenti* | DSM 11674 | Soy sauce mash |
| *S. cornubiensis* | DSM 105366 | Human cellulitis |
| *S. delphini* | DSM 20771 | Dolphin |
| *S. devriesei* | DSM 25293 | Cow teat |
| *S. edaphicus* | DSM 104441 | Black porous stone |
| *S. epidermidis* | NCTC 11047 | Human nose |
| *S. equorum* | DSM 20674 | Horse skin |
| *S. felis* | ATCC 49168 | Cat Ear |
| *S. gallinarum* | ATCC 35539 | Chicken nares |
| *S. haemolyticus* | ATCC 29970 | Human skin |
| *S. hominis* subsp*. hominis* | ATCC 27844 | Human skin |
| *S. hyicus* | ATCC 11249 | Pig with exudative epidermidis |
| *S. intermedius* | ATCC 29663 | Pigeon |
| *S. jettensis* | NCTC 13830 | Human blood |
| *S. kloosii* | DSM 20676 | Squirrel skin |
| *S. lugdunensis* | ATCC 43809 | Human axillary lymph node |
| *S. lutrae* | ATCC 700373 | Otter mammary gland |
| *S. massiliensis* | DSM 23764 | Human brain abscess |
| *S. microti* | DSM 22147 | Vole liver |
| *S. muscae* | DSM 7068 | Fly |
| *S. nepalensis* | DSM 15150 | Goat nose |
| *S. pasteuri* | DSM 10656 | Human vomit |
| *S. petrasii* subsp*. petrasii* | DSM 104505 | Human blood |
| *S. pettenkoferi* | DSM 19554 | Human blood |
| *S. piscifermentans* | ATCC 51136 | Fermented shrimp |
| *S. pseudintermedius* | DSM 21284 | Cat lung |
| *S. pseudoxylosus* | DSM 107950 | Bovine mastitis |
| *S. rostri* | DSM 21968 | Pig nose |
| *S. saccharolyticus* | DSM 20359 | Human plasma |
| *S. saprophyticus* subsp*. saprophyticus* | ATCC 15305 | Human urine |
| *S. saprophyticus* subsp*. bovis* | DSM 18669 | Bovine nose |
| *S. schleiferi* | ATCC 43808 | Human |
| *S. schweitzeri* | DSM 28300 | Monkey nose |
| *S. simiae* | DSM 17636 | Monkey faeces |
| *S. simulans* | ATCC 27848 | Human skin |
| *S. succinus* subsp*. succinus* | ATCC 700337 | Soil |
| *S. ureilyticus* | ATCC 49330 | Human skin |
| *S. warneri* | ATCC 27836 | Human skin |
| *S. xylosus* | ATCC 29971 | Human skin |
