# Supplementary material for: Evolutionary and Functional Analysis of Coagulase Positivity among the Staphylococci
Source: mSphere. 2021 Aug 4;6(4):e00381-21. doi: 10.1128/mSphere.00381-21 (PMC8386474; doi:10.1128/mSphere.00381-21)
Supplement: TABLE S2 [file msphere.00381-21-st002.docx]

TABLE S2. Genome sequences used in this study

| Species / subspecies | Strain | RefSeq assembly accession |
| --- | --- | --- |
| *M. fleurettii* | MBTS-1 | GCF_002018435 |
| *M. lentus* | NCTC 12102 | GCF_002902755 |
| *M. sciuri* subsp. *carnaticus* | CCUG 39509 | GCF_002902225 |
| *M. sciuri* subsp. *sciuri* | NCTC 12103 | GCF_002901825 |
| *M. stepanovicii* | DSM 26319 | GCF_002902525 |
| *M. vitulinus* | DSM 15615 | GCF_002902265 |
| *S. agnetis* | 908 | GCF_001442815 |
| *S. argensis* | DSM 29875 | GCF_002902305 |
| *S. argenteus* | MSHR1132 | GCF_000236925 |
| *S. arlettae* | NCTC 12413 | GCF_002902345 |
| *S. aureus* | Newman | GCF_000010465 |
| *S. auricularis* | DSM 20609 | GCF_001500315 |
| *S. caeli* | 82B | GCF_90097965 |
| *S. capitis* subsp. *capitis* | NCTC 11045 | GCF_002902325 |
| *S. capitis* subsp. *urealyticus* | DSM 6717 | GCF_002901925 |
| *S. caprae* | NCTC 12196 | GCF_002902725 |
| *S. carnosus* | DSM 11676 | GCF_002902605 |
| *S. casei* | DSM 15096 | GCF_002902445 |
| *S. chromogenes* | MU 970 | GCF_000696815 |
| *S. cohnii* | NCTC 11041 | GCF_002902365 |
| *S. coagulans* | M2043/98/1 | GCA_018885415 |
| *S. condimenti* | DSM 11674 | GCF_001618885 |
| *S. cornubiensis* | NW1 | GCF_900183575 |
| *S. croceilyticus* | CCUG 62728 | GCF_002902575 |
| *S. delphini* | 215100905101-2 | GCF_002369695 |
| *S. devriesei* | CCUG 58238 | GCF_002902625 |
| *S. edaphicus* | CCM 8730 | GCF_002614725 |
| *S. epidermidis* | NCTC 11047 | GCF_002901875 |
| *S. equorum* subsp. *equorum* | G8HB1 | GCF_000981135 |
| *S. equorum* subsp. *linens* | DSM 15097 | GCF_002901955 |
| *S. felis* | DSM 7377 | GCF_002902185 |
| *S. gallinarum* | DSM 20610 | GCF_000875895 |
| *S. haemolyticus* | NCTC 11042 | GCF_002901805 |
| *S. hominis* subsp. *hominis* | NCTC 11320 | GCF_002901845 |
| *S. hominis* subsp. *novobiosepticus* | CCUG 42399 | GCF_002902465 |
| *S. hyicus* | ATCC 11249 | GCF_000816085 |
| *S. intermedius* | NCTC 11048 | GCF_900458545 |
| *S. kloosii* | NCTC 12415 | GCF_002902055 |
| *S. lugdunensis* | NCTC 12217 | GCF_002901705 |
| *S. lutrae* | ATCC 700373 | GCF_002101335 |
| *S. massiliensis* | CCUG 55927 | GCF_002902705 |
| *S. microti* | DSM 22147 | GCF_002902635 |
| *S. muscae* | DSM 7068 | GCF_002901905 |
| *S. nepalensis* | DSM 15150 | GCF_002902745 |
| *S. pasteuri* | SP1 | GCF_000494875 |
| *S. petrasii* subsp. *jettensis* | CCUG 62657 | GCF_002902105 |
| *S. petrasii* subsp. *petrasii* | CCUG 62727 | GCF_002902565 |
| *S. pettenkoferi* | CCUG 51270 | GCF_002902685 |
| *S. piscifermentans* | NCTC 13836 | GCF_900186985 |
| *S. pseudintermedius* | ED99 | GCF_000189495 |
| *S. rostri* | DSM 21968 | GCF_002902145 |
| *S. saprophyticus* subsp. *bovis* | CCUG 38042 | GCF_002902545 |
| *S. saprophyticus* subsp. *saprophyticus* | ATCC 15305 | GCF_001997165 |
| *S. schleiferi* | DSM 6628 | GCF_002901995 |
| *S. schweitzeri* | FSCB1B | GCF_000751735 |
| *S. simiae* | CCUG 51256 | GCF_002902085 |
| *S. simulans* | NCTC 11046 | GCF_002902285 |
| *S. succinus* | DSM 14617 | GCF_001006765 |
| *S. ureilyticus* | DSM 6718 | GCF_002902235 |
| *S. warneri* | NCTC 11044 | GCF_002901765 |
| *S. xylosus* | NCTC 11043 | GCF_002901985 |
