# Supplementary material for: Evolutionary and Functional Analysis of Coagulase Positivity among the Staphylococci
Source: mSphere. 2021 Aug 4;6(4):e00381-21. doi: 10.1128/mSphere.00381-21 (PMC8386474; doi:10.1128/mSphere.00381-21)
Supplement: TABLE S3 [file msphere.00381-21-st003.docx]

TABLE S3. Average pairwise protein similarity between full-length vWbp and Coa sequences or the D1 and D2 prothrombin-binding domains

|  | Intra-clade | Hyicus clade | Intermedius clade | *S. condimenti* | *S. aureus* complex  (chromosome) | *S. aureus* complex  (SaPI-encoded) |
| --- | --- | --- | --- | --- | --- | --- |
| Full length protein: |  |  |  |  |  |  |
| Hyicus clade | 41.7% |  |  |  |  |  |
| Intermedius clade | 64.5% | 34.7% |  |  |  |  |
| *S. condimenti* | - | 35.1% | 45.5% |  |  |  |
| *S. aureus* complex (chromosome) | 73.2% | 34.5% | 40.3% | 43.7% |  |  |
| *S. aureus* complex (SaPI-encoded) | 79.3% | 36.0% | 38.0% | 40.1% | 61.7% |  |
| *S. aureus* complex (Coa) | 69.2% | 22.6% | 21.6% | 22.8% | 22.4% | 23.2% |

D1 and D2 domains:

| Hyicus clade | 43.8% |  |  |  |  |  |
| --- | --- | --- | --- | --- | --- | --- |
| Intermedius clade | 65.2% | 40.8% |  |  |  |  |
| *S. condimenti* | - | 38.9% | 53.0% |  |  |  |
| *S. aureus* complex (chromosome) | 55.5% | 40.0% | 46.2% | 46.6% |  |  |
| *S. aureus* complex (SaPI-encoded) | 68.6% | 42.2% | 43.5% | 41.6% | 46.8% |  |
| *S. aureus* complex (Coa) | 63.6% | 26.1% | 25.4% | 25.6% | 26.4% | 27.1% |
