# Supplementary material for: Evolutionary and Functional Analysis of Coagulase Positivity among the Staphylococci
Source: mSphere. 2021 Aug 4;6(4):e00381-21. doi: 10.1128/mSphere.00381-21 (PMC8386474; doi:10.1128/mSphere.00381-21)
Supplement: FIG S1 [file msphere.00381-21-sf001.docx]

**Fig. S1.** **50 µg ml^-1^** **recombinant vWbp proteins exhibit different host-specific coagulation phenotypes.** Coagulation of plasma from various hosts by 50 µg ml^-1^ recombinant protein of (A) *S. aureus* Newman Coa, (B) *S. aureus* Newman vWbp, (C) *S. intermedius* ATCC 29663 vWbp, (D) *S. pseudintermedius* ED99 vWbp, (E) *S. delphini* 8086 vWbp, and (F) *S. hyicus* ATCC 11249 vWbp. Coagulation was scored as 0.5 for trace levels of coagulation, 1.0 – 2.5 for partially coagulating plasma and 3.0 for a complete clot. Data is shown as the mean of 6 replicates with error bars representing standard deviation.
