# Supplementary material for: Evolutionary and Functional Analysis of Coagulase Positivity among the Staphylococci
Source: mSphere. 2021 Aug 4;6(4):e00381-21. doi: 10.1128/mSphere.00381-21 (PMC8386474; doi:10.1128/mSphere.00381-21)
Supplement: TABLE S4 [file msphere.00381-21-st004.docx]

TABLE S4. Primers used in this study

| Primer | Sequence (5’ to 3’) | Function |
| --- | --- | --- |
| *vwb* A | CCCGAATTCATGTCTCTGTTTAATTTTGTTC | pIMAY::*vwb* Construct |
| *vwb* B | TAATTCTACTCCCTTAATATGAATA | pIMAY::*vwb* Construct |
| *vwb* C | tattcatattaagggagtagaattaAATATCAAATAATCAAGAAAACC | pIMAY::*vwb* Construct |
| *vwb* D | CCCGAATTCTCTGTTCCCTCAGGCACCAC | pIMAY::*vwb* Construct |
| *vwb* Rep A | CCCGAATTCATGTCTCTGTTTAATTTTGTTC | pIMAY::*vwb* Rep Construct |
| *vwb* Rep B | CGACACAATGGCAGACGCTTCT | pIMAY::*vwb* Rep Construct |
| *vwb* Rep C | agaagcgtctgccattgtgtcgGGGGAGAAAAACCCTTATACATC | pIMAY::*vwb* Rep Construct |
| *vwb* Rep D | CCCGAATTCTCTGTTCCCTCAGGCACCAC | pIMAY::*vwb* Rep Construct |
| MCS F | TACATGTCAAGAATAAACTGCCAAAGC | Confirm Construct |
| MCS R | AATACCTGTGACGGAAGATCACTTCG | Confirm Construct |
| OUT F | TGGCGAAACATGGGCTACTTG | Confirm generation of Mutant Strains |
| OUT R | GTTTCGGCGCTTCTATCTGTTC | Confirm generation of Mutant Strains |
| Seq F | GGAGATTAAAAGTAATTTGTAACTA | Sanger sequencing |
| Seq R | CAACTTAAAATAAGGGTTTTC | Sanger sequencing |
| Mid F | ATACATCAGAACAATATAAAAAGAGC | Sanger sequencing |
| Mid R | ACAGATTGTTGTGGTGTTGC | Sanger sequencing |
| pET21b modi F | CCCGCTAGCATGACTGGTGG | Modify pET21b |
| pET12b modi R | CCCGCTAGCCTCCTTCTTAAAG | Modify pET21b |
| *coa* F | CCCGCTAGCATGATAGTAACAAAGGATTATAGTG | pET21b::coa |
| *coa* R | CCCTCGAGTTTTGTTACTCTAGGCCCATAT | pET21b::coa |
| *vwb* F | CCCGCTAGCATGGTGGTTTCTGGGGAGAAG | pET21b::*vwb* aureus |
| *vwb* R | CCCCTCGAGTTTGCCATTATATACTTTATTGATTTG | pET21b::*vwb* aureus |
| *vwb* F | CCCGCTAGCATGGTAGTAGACGTGAGAGAGAATCCA | pET21b::*vwb* hyicus |
| *vwb* R | CCCCTCGAGTTGACCATTATAAATTTTGTCGAT | pET21b::*vwb* hyicus |
| *vwb* F | CCCCATATGATCGTTACCGGGGAAGAAAATC | pET21b::*vwb* delphini |
| *vwb* R | CCCCTCGAGTTGACCATTGTAAGCTTTATCAAT | pET21b::*vwb* delphini |
| *vwb* F | CCCCATATGATAGTTACTGGGGAGAAAAAC | pET21b::*vwb* intermedius |
| *vwb* R | CCCCTCGAGTTGACCATTGTAAGCTTTATTAAT | pET21b::*vwb* intermedius |
| *vwb* F | CCCCATATGATTGTTTCGGGGGAGAAAAAC | pET21b::*vwb* pseudintermedius |
| *vwb* R | CCCCTCGAGTTGACCGTTGTAAGCTTTATTAAT | pET21b::*vwb* pseudintermedius |
| T7 F | TAATACGACTCACTATAGG | Confirm pET21b construct |
| T7 R | CGATCAATAACGAGTCGCC | Confirm pET21b construct |

Lowercase letters indicate complementary sequence to the relevant B cloning primer. Underlined sequences indicate restriction digestion sites.
